# Les mutuelles en Afrique : Enjeux et perspectives

**DOI:** 10.48327/mtsimagazine.n1.2021.78

**Published:** 2021-04-02

**Authors:** B. Boidin

**Affiliations:** CLERSÉ, Université de Lille, Lille, France

**Keywords:** CSU, Mutuelles, Afrique, Communauté, Participation, UHC, Mutual insurances, Africa, Community, Participation

## Abstract

Dès la fin des années 1970, la promotion de la communauté et de la participation des populations au système de santé renforcent l’idée que les mutuelles de santé sont des acteurs potentiellement majeurs de l’extension de la couverture contre les risques de santé. Mais l’histoire de la mutualité en Afrique a été rapidement parsemée d’embûches. Les plans d’ajustement structurel, l’augmentation de la contribution financière des usagers sous l’impulsion de l’initiative de Bamako, ont marqué un coup d’arrêt à l’esprit des mutuelles communautaires. Aujourd’hui les mutuelles sont de nouveau au cœur des initiatives internationales et nationales avec la promotion de la CSU (couverture sanitaire universelle). Mais elles doivent dépasser leurs carences historiques par la professionnalisation et l’obligation d’adhésion. Ce mouvement semble engagé tout en étant confronté au défi de préserver la place des communautés dans les systèmes de santé, principe fondateur du mouvement mutualiste.

## Abstract (en)

From the end of the 1970s, the promotion of the community and the participation of populations in the health system reinforced the idea that mutual health insurance was a potentially major player in the extension of coverage against health risks. But the history of mutuality in Africa was quickly strewn with pitfalls. The structural adjustment plans and the increase in the financial contribution of users under the impetus of the Bamako initiative put a stop to the spirit of community mutual insurances. Today, mutual insurances are once again at the heart of international and national initiatives with the promotion of the (UHC) Universal Health Coverage. But they must overcome their historical shortcomings through professionalization and compulsory membership. This movement seems to be underway, while at the same time facing the challenge of preserving the place of communities in health systems, a founding principle of the mutualist movement.

Alors que la promotion de la CSU depuis une dizaine d’années a fait des mutuelles un acteur central des politiques de santé en Afrique, il est utile de rechercher plus en amont les origines d’une telle montée en puissance mais aussi les obstacles restant à surmonter.

Dès la fin des années 1970, avec la déclaration d’Alma Ata de la « santé pour tous » (1978)^1^, puis la charte d’Ottawa (1986), la promotion de la communauté et de la participation des populations au système de santé renforcent l’idée que les mutuelles de santé sont des acteurs potentiellement majeurs de l’extension de la couverture contre les risques de santé. Fondées sur une vision démocratique et citoyenne, les mutuelles sont naturellement en phase avec des initiatives qui donnent un rôle important aux usagers.

Mais l’histoire de la mutualité en Afrique a été rapidement parsemée d’embûches. Alors que, dans de nombreux pays, le nombre de mutuelles de santé communautaire augmentait régulièrement à partir des années 1980, sous l’influence notamment d’Alma Ata et d’Ottawa, durant la même période les plans d’ajustement structurel ont bouleversé le paysage de la santé publique en Afrique en imposant une vision économiquement libérale de la santé publique, orientée vers la privatisation des services de santé et la contribution financière des populations. L’Initiative de Bamako lancée en 1987 était au départ axée sur la participation des usagers aux structures de santé mais la participation a finalement été réduite à sa dimension financière de paiement direct par les populations. Cette tendance a gravement nui à l’équité des systèmes de santé.

La remise en cause des plans d’ajustement structurel, le lancement des Objectif du Millénaire pour le développement puis des Objectifs de développement durable (avec la CSU comme l’une des cibles emblématiques), la promotion de la CSU comme moteur de la santé à l’échelle mondiale (inscrite dans une résolution des Nations unies en 2012) ont redonné au mouvement mutualiste et à la participation communautaire une place centrale dans les politiques de santé en Afrique. Pourtant, plusieurs obstacles majeurs continuent de se dresser contre la réalisation d’une véritable CSU portée par les mutuelles (Ridde et al. 2018^2^).

D’abord, la participation communautaire, que les acteurs de l’aide appelaient de leurs vœux, n’est toujours pas une réalité. Les usagers sont souvent peu impliqués dans les décisions des mutuelles malgré des dispositifs qui ont été lancés pour favoriser leur participation, tels que l’exemption de paiement ciblée, qui a permis de réduire les inégalités mais sans améliorer l’inclusion des populations en tant qu’acteurs du système de santé.

Ensuite, le succès des mutuelles locales reste très mitigé car il repose sur le volontarisme et se heurte à de nombreuses barrières à l’adhésion qui ont été largement documentées (Ridde et al., 2010; Waelkens et al., 2017^3^): faible capacité contributive des populations, prise en compte très inégale des besoins selon les mutuelles et les pays (les paniers de soins associés à la couverture sont de qualité et de nature hétérogène), faible prise en compte des besoins des plus pauvres, etc.

Enfin, les mutuelles communautaires souffrent d’une gestion défaillante liée au caractère bénévole de leur gestion. Le statut de bénévole ou de quasi-bénévole (assorti d’une petite indemnité) des gérants entraîne des problèmes financiers récurrents et une absence de conservation des données financières d’une année à l’autre (Dupré, 2010^4^).

Au total, le mouvement mutualise en Afrique, considéré comme un fondement essentiel de l’avancement vers la CSU, ne parvient pas encore à surmonter les obstacles à la fois techniques, financiers, humains, organisationnels et institutionnels qui nuisent à sa montée en puissance. C’est la raison pour laquelle l’appui de tous les acteurs de la santé et de la coopération internationale, au premier chef le mouvement mutualiste international, est une priorité. Car malgré les efforts des organisations de solidarité internationale, des gouvernements, des organisations internationales et des partenaires techniques et financiers, ce qui manque le plus aux structures mutualistes en Afrique est de bénéficier des compétences et de l’expérience de leurs pairs, à la condition bien sûr que soient prises en compte les spécificités culturelles et historiques locales, afin de ne pas se contenter de dupliquer des « modèles voyageurs » (normes internationales plaquées sur différents systèmes locaux, cf. Olivier de Sardan, 2019^5^).

Comment les mutuelles de santé non africaines pourraient-elles contribuer au succès du mouvement mutualiste en Afrique ? D’abord, en tirant les leçons des échecs et obstacles précédents, comme l’illustrent les autres articles de ce dossier à travers leurs préconisations. Les enseignements des expériences récentes montrent que les mutuelles ont été promues dans une optique largement volontariste, bénévole et non obligatoire. Or il semble exister aujourd’hui un consensus (Ridde et al., 2018^6^) sur le fait qu’une extension viable des mutuelles en vue de la CSU repose non seulement sur la professionnalisation des gérants des mutuelles, mais également sur l’obligation d’adhésion, même si des contraintes fortes (financières, humaines) doivent être dépassées pour respecter ces deux conditions.

En ce qui concerne la professionnalisation des mutuelles, certains pays ont suivi ce chemin avec un certain succès (le Rwanda, avec des mutuelles gérées de façon croissante par des professionnels de la fonction publique) où semblent s’orienter dans cette direction (Mali, Sénégal). Mais cela n’est pas sans poser des problèmes car la professionnalisation a pour corollaire un contrôle plus élevé de l’État qui veut disposer d’un droit de regard voire de régulation sur les mutuelles. Évidemment cela semble a priori contraire au principe d’indépendance des mutuelles et surtout de participation accrue des populations. Un équilibre devrait être trouvé en préservant les caractéristiques fondamentales des mutuelles.

S’agissant de l’obligation d’adhésion, l’idée a fait son chemin dans plusieurs pays africains. Elle est soutenue par l’OMS (Mathauer et al. 2017^7^). Après le Rwanda, le Ghana fait figure de précurseur, avec certes un succès plus mitigé. Mais ce qui ressort surtout des expériences ayant pour but de rendre obligatoire l’adhésion des populations est que, là encore, des choix socio-politiques sont inévitables, entre d’une part, promouvoir la participation communautaire qui est une visée bien légitime, d’autre part, assurer l’universalité de la couverture maladie par des actions publiques volontaristes et, parfois, de reprise en main de la CSU à travers le contrôle plus ou moins étendu des financements et de la gouvernance des mutuelles (Ridde et al. 2018).

Si la professionnalisation et l’obligation d’adhésion font aujourd’hui figure de conditions favorables à l’atteinte de la CSU en Afrique, une question toujours d’actualité est donc de savoir comment conduire ces évolutions tout en restant fidèle aux principes mutualistes de participation communautaire. Cette question trouve une acuité toute particulière en Afrique où, précisément, le caractère démocratique des mutuelles reste dans certains cas à construire (Alenda, Boidin, 2019^8^) avec une faible participation des adhérents aux décisions. Les directives internationales ont fait la promotion des mutuelles communautaires mais le chemin pris depuis quelques années rend floue la place du qualificatif « communautaire » dans la CSU. Enfin, dans ce contexte d’influence permanente de dispositifs apportés de l’extérieur, on peut s’interroger sur les capacités d’élaboration de programmes originaux par les Pouvoirs publics locaux. L’un des grands défis du mouvement mutualiste international est alors de soutenir le développement des mutuelles africaines dans l’extension de la CSU, tout en préservant les spécificités locales des mutuelles de santé.

## Conflits D'intérêts

L’auteur ne déclare aucun conflit d’intérêt.
